# Suppressive Effects of Rosa Damascena Essential Oil on Naloxone- Precipitated Morphine Withdrawal Signs in Male Mice

**Published:** 2013

**Authors:** Navid Abbasi Maleki, Saeid Abbasi Maleki, Reza Bekhradi

**Affiliations:** a *Departement of Pharmacology, Urmia Branch, Islamic Azad University, Urmia, Iran.*; b *Research Development and Training Center, Barij Essence Pharmaceutical CO, Kashan, Iran. *

**Keywords:** Rosa damascene, Essential oil, Morphine withdrawal, GABAergic system, Mice

## Abstract

This research was done to test the effect of Rosa damascena essential oil on withdrawal signs of naloxone-precipitated morphine in male mice. Morphine dependence was induced by injection (IP) three times daily at doses of 50, 50 and 75 mg/kg, respectively, for 3 days. On day 4, after the last administration of morphine, Rosa damascena essential oil was administered at different concentrations (5, 2 and 40%, IP) 30 min before administration of naloxone (5 mg/kg, IP). The following actions were taken as signs of withdrawal and records taken for jumping as a number and scores of 0 to 3 were given for incidences of grooming, teeth chattering, rearing, writing, diarrhea, wet dog shakes and climbing during a 30 min period**. **Results showed that different concentrations of Rosa damascena essential oil significantly reduced signs of morphine withdrawal compared to the control group in terms of number of jumps (p < 0.05 and p < 0.01), grooming, teeth chattering, rearing, climbing, wet dog shakes and writhing, but not for diarrhea (p < 0.05). In conclusion it seems that GABAergic activity induced by flavonoids from Rosa damascena essential oil can alleviate signs of morphine withdrawal, but further studies need to be done to better understand this mechanism.

## Introduction

Rosa damascene is a plant belonging to the Rosaceae family, commonly known as Damask rose, in Iran it is known as Gole Mohammadi (1). The flowers of this plant are large, showy and colorful (6). It is cultivated for decorative purposes and for its scent (2). In Iran, it is also cultivated for the production of essential oil and rose water (3). The *R. damascena *plant has also been used for medicinal applications and several *in-vivo *and *in-vitro *studies have been done on various products and isolated constituents from its flowers, petals and hips (seed-pot) (4). This plant contains several compounds such as terpenes, glycosides, flavonoids, anthocyanins, carboxylic acid, myrcene, vitamin C, kaempferol, quarcetin, and geraniol (4-6). It is well known that Rosa inhibits reactivity of the hypothalamus and pituitary systems in rats and can suppress activity of the central nervous system (2). Research has demonstrated that Rosa damascena has anti-HIV, antioxidant, antitussive, antispasmodic, sedative, hypnotic, anticonvulsant and laxative properties as well as prokinetic and antidepressant effects (3, 7-11). 

Chronic use of opiate substances induces tolerance and dependence so that any abrupt cessation of opiate administration results in the user experiencing withdrawal syndrome (12). Congenitally, methadone, a *μ *receptor agonist, has been used to alleviate these withdrawal signs (13). However methadone also produces its own side effects and withdrawal symptoms (14). Some antidepressants and other non-opiate substances have been developed and used to prevent withdrawal syndrome. Classical antidepressants such as fluvoxamine or sertraline can reduce opioid withdrawal syndrome (15). According to the antidepressant activity of different concentrations (5, 20 and 40%) of Rosa damascena (11), it can be hypothesized that the essential oil has a therapeutic effect, especially for reducing effects of withdrawal syndrome in drug-addicted patients. This study aimed to determine whether essential oil from Rosa damascena has a suppressive effect on withdrawal signs in morphine dependent mice.

## Experiment

*Animals*

Male albino mice (Pasteur Institute, Tehran, Iran), weighing 20-30 g at the beginning of the experiment were used for these tests. Animals were housed eight per cage, maintained at 23± 1°C with a controlled 12 h light/dark cycle and with ad libitum food and water. Each mouse was used only once and each treatment group consistent of eight animals. They were acclimatized to the conditions of the experiment for at least 1-week prior to use. All procedures were performed during the light phase and done in accordance with guidelines authorized by the School of Medicine at Tehran University of Medical Science.

*Drugs*

The following drugs were used for the tests: morphine sulfate (Temad, Iran), Naloxone HCL (Tolidaru, Iran) and Diazepam HCL (Abidi Pharmaceutical Co, Iran). All drugs were dissolved in saline and were injected intraperitoneally (IP) at volumes of 10 mL/kg.

*Preparation of the essential oil of Rosa damascena*

Rosa damascena flowers were collected from Kashan (central Iran) in spring 2011. The plant species was identified by botanical specialists of the biological department of Barij Essence Pharmaceutical Co (Kashan, Iran). Essential oil was obtained from fresh flowers taken from the plant by stem distillation (hydro-distilled) at the Barij Essence Pharmaceutical Co and stored in a refrigerator (- 4**°**C). To administer the essence it was reconstituted in normal sterile saline and filtered before injection (using a bacterial filter). 

*Induction of morphine dependence*

Morphine was injected (IP) three times daily (8.00, 12.00 and 16.00) at doses of 50, 50 and 75 mg/kg, respectively, for 3 days. On day 4, only a single morning dose of morphine (50 mg/kg) was injected before the naloxone injection (16).

*Observation of morphine withdrawal*

Withdrawal signs were precipitated by injection of naloxone (5 mg/kg, IP) 2h after the injection of morphine. Following the naloxone injection, animals were immediately placed individually on filter paper in a clear plastic cylinder (15 cm in diameter and 50 cm in height) for observation. The behavior of each animal was recorded for 30 minutes using a digital camera. Evaluations were taken for the following signs: jumping, climbing, wet dog shakes, grooming, teeth chattering, diarrhea, rearing and writhing. These signs were rated every 10 min using a scale from 0 to 3: A score of 0 indicated that a sign was absent during the 10 min observation period; a score of 1 indicated low intensity behavior; a score of 2 indicated average intensity and a score of 3 showed maximum intensity. Incidence of diarrhea was evaluated every 20 min and given a score accordingly (17).

*Grouping of animals and administration of extracts*

Forty mice were randomly divided in to groups of eight. Morphine was administered to mice in all groups as discussed above. 

I: Control group (as a negative group): In this group a solvent of rose essential oil (normal saline and tween 80) was administered IP 30 min after the last dose of morphine and 30 min later, naloxone was injected.

II: Diazepam group (as a positive group): In this group diazepam (5 mg/kg) was administered IP 30 min after the last dose of morphine and 30 min later, naloxone was injected.

 III: Rosa damascene essential oil group: In this group different concentrations of Rosa damascena essential oil (5, 20 and 40%) were administered IP 30 min after the last dose of morphine and 30 min later, naloxone was injected. 

*Statistical analysis*

Data were expressed as mean ± SEM for eight mice in each group. One-way AONOVA followed by the Tukey test was used for comparisons of data. The Mann-Whitney U test was used for comparisons of checked signs data versus the control group. Difference of p < 0.05 was considered statically significant. 

## Results

**Figure 1:**
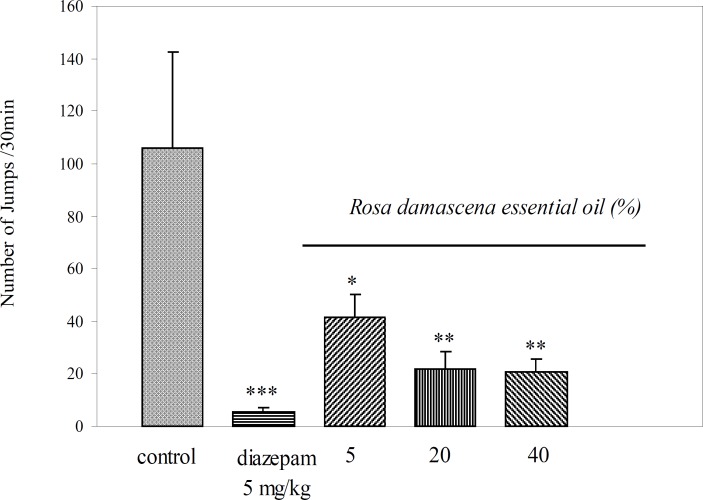
Effects of different concentrations of *Rosa damascena *essential oil on naloxone- precipitated jumping in morphine-dependent mice (n = 8, Mean ± SEM, ^* ^p < 0.05, ^** ^p < 0.01 and ^***^ p < 0.001 compared to control group, Tukey-Karamer test).

Results showed that intraperitoneal administration of Rosa damascena essential oil at concentrations of 5, 20 and 40% significantly, according to the dose administered, reduced the number of jumping episodes in morphine dependent mice (p = 0.012, p = 0.005 and p = 0.002, respectively (Figure 1)). Diazepam, as a reference drug caused a significantly reduced number of jumping episodes (p= 0.000, (Figure 1)). Essential oil at all concentrations significantly decreased wet dog shake (p = 0.037, p = 0.006 and p = 0.044, respectively (Table 1)), writhing (p = 0.008, p = 0.008 and p = 0.024, respectively (Table 1) , climbing (p = 0.034, p = 0.034 and p = 0.021, respectively (Table 1) and grooming (p = 0.014, p = 0.014 and p = 0.008, respectively (Table 1)).Rearing and teeth chattering decreased only at a higher concentration (40%) of essential oil (p = 0.039 and p = 0.000, respectively (Table 1)). Different doses of essential oil haven’t any significant effect on decreasing of diarrhea. Diazepam group compared to control group significantly reduced other signs such as grooming (p = 0.008), teeth chattering (p = 0.012), climbing (p = 0.034), rearing (p = 0.042) wet dog shakes (p = 0.028) writhing (p = 0.008) and diarrhea (p = 0.019) (Table 1). 

**Table 1:** Effects of different concentrations of *Rosa damascena *essential oil on checked signs of morphine withdrawal signs

**Diarrhea**	**writhing**	**Wet dogshakes **	**Rearing**	**Climbing**	**Teethchattering**	**Grooming**	**Treatment**
3 (3-2)	5.1 (2-1)	2 (75.2-1)	5.1 (3-1)	2 (2-25.1)	2 (75.2-2)	5.2(3-2)	Control
1* (75.1-1)	0* (75.0-0)	1 * (1-0)	5.0* (1-0)	1 * (1-0)	5.0* (75.1-0)	5.0*(1-0)	Diazepam5 mg/kg
3 (3-3)	0* (75.0-0)	1* (1-25.0)	1 (1-1)	1* (1-0)	1 (75.1-1)	1*(1-1)	Rosa Damascena5%
2.5 (3-2)	0* (75.0-0)	5.0* (1-0)	1 (75.1-75.0)	1* (1-0)	1 (1-25.0)	1*(1-1)	Rosa Damascena20%
2 (2-5.0)	5.0* (1-0)	1* (1-1)	0* (1-0)	5.0* (1-0)	0* (0-0)	5.0*(1-0)	Rosa Damascena40%

## Discussion

The results of this study indicate that Rosa damascena essential oil has component(s) that could attenuate the signs of morphine withdrawal in morphine dependent mice. Jumping is one of the most common signs used to assess the severity of morphine withdrawal. In agreement with our data, another study reported that Rosa damascena has laxative and prokinetic effects; thus applied doses of essential oil haven’t any significant effect in terms of decreasing incidence of diarrhea (8). Other research has reported similar responses to the results of this study in terms of suppressed behavior for wet dog shake, writhing, climbing, grooming, rearing, teeth chattering and diarrhea from diazepam (18). 

The family Rosaceae is a well known traditional folk medicine used for treating nervous system breakdown (19). Rosa damascena contains several components such as flavonoids, kaempferol, geraniol, citranellol, eugenol, linalool, nerol, myercene and vitamin C that have pharmacological effects on the CNS (6). It has been demonstrated that flavonoids and kaempferol of Rosa damascena have anxiolytic and/or antidepressant effects that have been ascribed to their affinity to affect the central benzodiazepine receptors (20) and it has been reported that flavonoids can suppress opioid withdrawal syndrome (21). Furthermore, studies have shown that co-administration of opiates with antidepressants has some benefits such as an additive effect on analgesic activity (22); an ability to reduce the adverse effects of opiates by producing an opioid-sparing effect (23) and by reducing potential harm from the use of opiates both directly by having a rewarding effect, or, indirectly by improving a depressed mood experienced from opiate withdrawal (24). Results of this study are also in accordance with other research demonstrating that Rosa damascena essential oil with GABAergic activities acts as an antidepressant. Moreover, hypnotic and anticonvulsant effects of Rosa damascena essential oil attribute to its affinity with the GABAA- system (9, 25). These effects mean that Rosa damascena essential oil can reduce the severity of symptoms of morphine withdrawal. Results of these tests demonstrate that both GABA_A_ and GABA_B_ receptor agonists are involved in signs induced by morphine withdrawal. For example, several studies have reported that baclofen (GABA_B_ agonist) and muscimol (GABA_A_ agonist), both inhibited naloxone induced jumping behavior in morphine dependent mice in a dose-dependent manner (26, 27). 

In conclusion, it seems that flavonoids of Rosa damascena essential oil with GABAergic activity can attenuate morphine withdrawal signs. But further studies need to be carried out to better understand this mechanism.
